# Shear Bond Strength of Self-Adhering Flowable Composite and Resin-modified Glass Ionomer to Two Pulp Capping Materials

**DOI:** 10.22037/iej.2017.21

**Published:** 2017

**Authors:** Maryam Doozaneh, Fatemeh Koohpeima, Maryam Firouzmandi, Forugh Abbassiyan

**Affiliations:** a *Department of Operative Dentistry, Dental School, Shiraz University of Medical Science, Shiraz, Iran; *; b *Dental School, Shiraz University of Medical Science, Shiraz, Iran*

**Keywords:** Pulp Capping Agent, Self-Adhering Flowable Composite, Shear Bond Strength

## Abstract

**Introduction:**

The aim of this study was to compare the shear bond strength of a self-adhering flowable composite (SAFC) and resin-modified glass ionomer (RMGI) to mineral trioxide aggregate (MTA) and calcium-enriched mixture (CEM) cement.

**Methods and Materials:**

A total of 72 acrylic blocks with a central hole (4 mm in diameter and 2 mm in depth) were prepared. The holes were filled with MTA (sub group A) and CEM cement. The specimens of both restorative materials were divided into 6 groups; overall there were 12 groups. In groups 1 and 4, SAFC was used without bonding while in groups 2 and 5 SAFC was used with bonding agent. In all these groups the material was placed into the plastic mold and light cured. In groups 3 and 6, after surface conditioning with poly acrylic acid and rinsing, RMGI was placed into the mold and photo polymerized. After 24 h, the shear bond strength values were measured and fracture patterns were examined by a stereomicroscope. Data were analyzed using the two-way ANOVA and student’s t-test.

**Results:**

The use of bonding agent significantly increased the shear bond strength of FC to MTA and CEM cement (*P*=0.008 and 0.00, respectively). In both materials, RMGI had the lowest shear bond strength values (2.25 Mpa in MTA and 1.32 Mpa in CEM). The mean shear bond strength were significantly higher in MTA specimen than CEM cement (*P*=0.003). There was a significant differences between fracture patterns among groups (*P*=0.001). Most failures were adhesive/mix in MTA specimen but in CEM cement groups the cohesive failures were observed in most of the samples.

**Conclusion:**

The bond strength of self-adhering flowable composite resin to MTA and CEM cement was higher than RMGI which was improved after the additional application of adhesive.

## Introduction

Following traumatic injuries or restorative procedure, normal dental pulp exposure may occur inadvertently. In this situation, vital pulp therapy (VPT) is performed by placing the direct pulp capping biocompatible materials to maintain the health and vitality of dental pulp [1]. It is obvious that the success of such treatment is related to seal the pathways between the root canal and surrounding tissues [2]. Therefore, the ideal bonding between pulp capping agents and restorative materials is essential [3]. Mineral trioxide aggregate (MTA) is a commonly used material for VPT. After its setting, MTA forms calcium hydroxide in the presence of tissue fluids, which creates the antibacterial environment [4]. It also encourages differentiation and migration of hard tissue cells that release the hydroxyapatite on surface and produce the biological seal [5, 6]. 

Calcium-enriched mixture (CEM) cement is suggested biocompatible pulp capping material produced to overcome the drawbacks of MTA [7]. These biomaterial contains some superior physical and antibacterial properties compared with MTA [8]. 

After VPT a definite leakage free restoration (composite or amalgam) should be used. Different studies showed that acid etching before composite build up and nature of solvent in the adhesives may influence the mechanical properties and bond strength of pulp covering agent to composite resin [2, 9]. Hashem *et al. *[10] also demonstrated that the coronal restorations over pulp capping agents should have low condensation forces. The use of an intermediate restorative materials or liner has been emphasized between the pulp capping agent and final restorative materials. Resin modified glass ionomer (RMGI) and flowable composite (FC) may be suitable materials to be used over the pulp capping agents because of low condensation force required for their placement. The creation of proper bond between intermediate materials and pulp capping agents are essential to seal and guarantee the success of treatment [11]. Oskoee *et al. *[12] and Ajami *et al*. [13] showed that the highest shear bond strength was observed when composite resin was bonded to RMGI than MTA or CEM cement and suggested that it is advisable to cover these pulp capping materials with RMGI before composite buildup.

Vertise Flow (VF; Kerr, orange, CA, USA) is a self-adhering flowable composite which combines an all-in-one adhesive system and a flowable composite for a step-less system [14]. The presence of glycerol phosphate dimethacrylate monomer (GPDM) in this SAFC eliminates a separate adhesive application and save chair time and handling errors. Vichi *et al. *[15] revealed that this composite had lower bond strength to enamel and dentin but better marginal sealing was observed in comparison with the other all-in-one adhesive systems

There is no information on the adhesion of SAFC to MTA or CEM cement. Therefore, the purposes of this study was to determine the shear bond strength of SAFC alone and in conjunction with a self-etch adhesive to MTA and CEM cement and also compare it with RMGI cement. The null hypothesis was that there would be no differences between the bond strength values in different study groups.

## Materials and Methods

In this study, 72 acrylic blocks (Acropars, Tehran, Iran) were with a central hole measuring 4 mm in diameter and 2 mm in depth. The samples were divided into two groups (*n*=36). RootMTA (Tabriz, Iran) and CEM (Bionique Dent, Tehran, Iran) were mixed according to their manufacturers’ instructions. The pastes were poured into the holes in the center of acrylic blocks, flattened with a spatula, covered with a moist cotton pellet and temporary filling materials (Cavisol, Golchai Co., Tehran, Iran). Then, the specimens were stored for 72 h at 37^°^C temperature and 100% humidity. After storage, the temporary materials and cotton pellets were removed without interfering with the surfaces of the pulp capping materials. Then, the specimens of each material were divided into the six groups (*n=12*). 

In groups 1 and 4, after air drying of the specimen, the SAFC (Vertise Flow, Kerr, orange, CA, USA) was actively applied directly over MTA or CEM with no adhesive. The SAFC was placed into the plastic mold with 3 mm diameter and 2 mm height in one increment. Then the SAFC was light cured with light density 600 mw/cm ^2^(VIP junior; Bisco, Schumburg, USA) for 40 sec. 

In groups 2 and 5, after air drying of the specimen, Opti-Bond all-in-one adhesive (Kerr Dental, orange CA, USA) was actively applied on the surfaces with a brush and after air thinning, was light cured for 20sec. Then SAFC was subsequently applied to the conditioned surfaces, similar to the previous groups.

In groups 3 and 6, the surfaces of pulp capping agents were conditioned with 10% poly acrylic acid (GC Corp. Tokyo, Japan) for 20 sec, then rinsed for 30 sec and air dried. After that, the powder and liquid of RMGI (Fuji II Lc; GC Corp.; Tokyo, Japan) was mixed according to the manufacturer’s instructions; the paste was placed into the plastic mold and light cured for 40 sec similar to above mentioned groups.

The prepared specimens were kept in 100% relative humidity at 37^°^C temperature for 24 h. After that, the plastic molds were carefully removed from the specimen before the shear bond strength test.

The specimens were mounted in the universal testing machine (Instron; Zwich, Germany) and shear forces were applied at the crosshead speed of 0.5 mm/min until the fracture occurred. The maximum loads at failure were recorded in Newton and were then converted into the Mps.

The fractured specimens were observed under a stereomicroscope under 40× magnification to determine the failure mode. The failure types were categorize as adhesive (two flat surfaces, showing failure of filling materials/pulp capping bond), cohesive (any deficiency in the pulp capping agent surface) and mixed (combination the adhesive residue and deficiency in the pulp capping surfaces).

The two-way ANOVA analysis was applied to determine the interaction effect between the experimental groups and if it was applicable, the post hoc comparison and t-test was used to compare the shear bond strength results among groups. Also, the chi-square and Fissure’s exact test were used to compare the fracture surface pattern between groups. The level of significance was set at 0.05.

## Results

Table 1 shows the mean and standard deviations of shear bond strength for all groups. There was a significant interaction effect between the intermediate filling materials (SAFC and RMGI) and the pulp capping agents (Root MTA and CEM cement). The use of an all-in-one adhesive system significantly increased the shear bond strength of SAFC to MTA and CEM cement (*P*=0.008, 0.000, respectively). In both pulp capping agents, RMGI had the lowest shear bond strength values (*P*=0.05). For all intermediate materials in this study, the mean shear bond strength values were significantly higher in MTA than CEM; however, these difference was not obvious in RMGI than other intermediate materials (*P*=0.003). There were a significant difference between fracture patterns between groups (*P*=0.001). Most failures in MTA specimens were adhesive/mix, but in CEM cement specimens, the cohesive failure was the predominant mode of fracture except for RMGI materials which adhesive failures were also observed. All groups showed significant differences with CEM/SAFC with no bonding (Table 1).

## Discussion

The results of our study showed that SAFC (with or without application of adhesive) had superior bond strength compared to RMGI, either over MTA or CEM cement. The null hypothesis was rejected, because the bond strength changed in relation to adhesive application and filling materials. 

More researches have shown that, both MTA and CEM cement can be used effectively as pulp capping agents because they have the ability to stimulate the differentiation of dental pulp stem cells to odontoblast like cells and ultimately initiate the formation of dentinal bridge which is thicker, less porous along with less pulp inflammation than the calcium hydroxide material [6, 16, 17]. 

In this study, we used SAFC as an intermediate material before permanent composite restoration over Root MTA or CEM cement. The SAFC used in this experiments a novel flowable resin based composite that eliminates the etching, priming and bonding steps in order to simplify the adhesive procedures to dentin and enamel [15]. 

The results of our study demonstrated that surface treatment with an all-in-one adhesive before SAFC significantly increased the shear bond strength of SAFC to Root MTA and CEM cement. It was in consistent with the results of the study by Tuloglu *et al. *[18]. Also, the study by Neelakantan *et al*. [19] showed that one-step-self etching adhesive demonstrated the strongest bond to white MTA after 24 h compared to total etching or two-step self-etching adhesives. This can be attributed to the sensitivity of MTA and other calcium silicate cements to acidic environment [20]. However, the study by Bayrak *et al. *[2] indicated that etch-and-rinse adhesives showed stronger bond than self-etch adhesive were adhesive for bonding composite resin to white MTA. It is demonstrated that the different bonding systems (total-etch *vs.* self-etch) did not have a significant effect on the shear bond strength of composite resin to pulp capping biomaterials [21, 22]. Kayahan *et al. *[9], evaluated the effect of acid etching on the compressive strength of calcium-silicate based materials and concluded that the acid etching significantly reduced the SBS of Angelus MTA and CEM cement. Several studies have reported that the acidic environment would reduce the several physical properties of MTA [9, 23]. In SEM evaluation, the disordered structure, selective dissolution and detachment of filler particles were observed after acid etching process [24]. In our study, the pH value for SAFC and bonding system were reported as 1.9 by the manufacturer; therefore, both acted as a mild self-etch adhesive over the pulp capping agents [15]. Thus, it did not cause the adverse effect of highly acidic pH on MTA or CEM cement.

As mentioned previously, the additional application of self-etching bonding would enhance the shear bond strength of SAFC to MTA and CEM cement. However, the results were in argue with Yesilyurk *et al. *[25] who concluded that the self-etch adhesive significantly reduced the shear bond strength of SAFC to Bioaggregate. However, the surface treatment of Bioaggregate with an etch-and-rinse adhesive significantly increased the shear bond strength after 72 h [25]. In our study, we used Opti Bond all-in-one as a self-etch adhesive system along with SAFC which has the same adhesive technology and chemical composition to eliminate the probable adhesive interaction of two different technology used in Yesilyurk’s study. The second possible reason for enhancement of Opti Bond self-etch adhesive over calcium silicate based cements, is that it contains ethanol, acetone and water in its composition as solvents. Hence, smaller contact angle and better wettability of the MTA and CEM cement would be expected [19]. This factor plus the lower viscosity of Opti Bond self-etch adhesive may be responsible for more macromechanical retention than high viscose SAFC, which is the principle adhesion mechanism for bonding of the pulp capping agent/flowable composite [25].

**Table 1:** Mean (SD) of shear bond strength for experimental groups

	**Shear Bond Strength (Mpa)**	**Type of failure mode N (%)**		
		**Adhesive failure**	**Cohesive failure**	**Mixed failure**
**MTA+SAFC**	7.30 (1.33)	2 (16.7)	3 (25)	7 (58.3)
**MTA+SAFC (bonded)**	8.80 (1.24)	4 (33.3)	2 (16.7)	6 (50)
**MTA+RMGI**	2.25 (0.77)	4 (33.3)	3 (25)	5 (41.7)
**CEM+SAFC**	3.50 (1.25)	0	12 (100)	0
**CEM+SAFC (bonded)**	5.94 (1.09)	1 (8.3)	7 (58.3)	4 (33.3)
**CEM+RMGI**	1.32 (0.61)	5 (41.7)	5 (41.7)	2 (16.7)

In this study, SAFC had significantly higher mean of shear bond strength values than RMGI for both MTA and CEM cement. This results were contrary to the results reported by Ajami *et al. *[21] that showed superior bond of composite compared to RMGI to three pulp capping agents. In this study, 10% polyacrylic acid conditioner was used before applying RMGI. Acidic content and rinsing may be one reason for reducing the shear bond strength of RMGI to pulp capping materials. However, Gulati *et al. *[26] revealed that conditioning MTA with 10% polyacrylic acid could not significantly affect the shear bond strength of RMGI.

According to the manufacturers, SAFC used in this study consists of phosphate functional monomer (GPDM) which may interact with the calcium ions [27]. Both MTA and CEM cement have different calcium compounds which release calcium hydroxide during and after setting [28, 29]. The possible chemical bond with GPDM of SAFC and MTA and CEM cement may responsible for the higher shear bond strength than RMGI that should be analyzed in further researches. In the present study, MTA had significantly higher shear bond strength than CEM cement for both filling materials. Sobhnamayan *et al. *[30] revealed that even after 72 h, CEM cement was not completely hardened and had a semi-hard consistency in contrast to MTA. These results confirm the failure mode analysis obtained in the present study, where most of the failures observed in the CEM cement were cohesive, in contrast to MTA and RMGI in which adhesive failures were seen the most. Ajami *et al. *[21] reported that the shear bond strength of resin composite to MTA was weaker than CEM cement and NMTA. The chemical composition and different shape, size and distribution of the hydroxyapatite crystals may explain the different behavior of these two materials [7, 31]. In this study a 72-h delay time in 100% humidity was chosen for setting of MTA and CEM cement because many studies suggested that three days are required for displacement resistance and complete setting of MTA [3, 32]. Bond strength tests are the most common laboratory test for analyzing the performance of adhesive restoration [33]. Because MTA and CEM cement are brittle materials, the shear bond strength test was chosen and considered as the best method for analysis in our study [34]. Further scanning electron microscope (SEM) and transmission electron microscope (TEM) studies should be planned to analyze the possible bond between the self-etching FC and different pulp capping agents in different time intervals in long-term storage and especially in clinical set-ups.

## Conclusion

Within the limitation of this *in vitro* study, it was found that the application of a self-etch adhesive improves the bond quality of Vertise Flow to MTA and CEM cement. Moreover, the bond strength of Vertise Flow was significantly greater than resin-modified-glass ionomer in both materials. All materials had significantly higher bond strength in MTA group than CEM cement after 72 h setting.
